# Alleviation role of exogenous cadaverine on cell cycle, endogenous polyamines amounts and biochemical enzyme changes in barley seedlings under drought stress

**DOI:** 10.1038/s41598-023-44795-z

**Published:** 2023-10-15

**Authors:** Serkan Ozmen, Selma Tabur, Signem Oney-Birol

**Affiliations:** 1 Department of Biology, Faculty of Arts and Sciences, Süleyman Demirel University, 32260 Isparta, Turkey; 2 Department of Moleculer Biology and Genetics, Faculty of Arts and Sciences, Burdur Mehmet Akif Ersoy University, 15030 Burdur, Turkey

**Keywords:** Enzymes, Hormones, Cell biology, Plant sciences, Climate sciences

## Abstract

Cadaverine (Cad), which has an independent synthesis pathway compared to other polyamine (PA) types, contributes to the health of plants by regulating plant growth and development, abiotic stress tolerance and antioxidant defense mechanisms. In this work, experiments were carried out to understand the effects of exogenous Cad (10 µM) application under drought stress (%22 PEG 6000) and without stress on cell cycle, total protein content, endogenous PA levels, and biochemical enzyme activities in barley (*Hordeum vulgare* cv. Burakbey) considering the potential of Cad to stimulate the drought-related tolerance system. Cad application in a stress-free environment showed an effect almost like low-impact drought stress, causing changes in all parameters examined compared to samples grown in distilled water environment (Control). The results clearly show that Cad applied against the negative effects of drought stress on all parameters creates a drought resistance mechanism of the plant. Accordingly, Cad applied together with drought stress increased the density of cells in the cell cycle (G1–S and S–G2 phases) and the amount of endogenous (spermidine 10% and spermine 40%) PAs. In addition, while superoxide dismutase (SOD) (5%), (CAT) (55%) and ascorbate peroxidase (APX) (18%) enzyme levels increased, a stress response mechanism occurred due to the decrease in total protein content (20%) and malondialdehyde (MDA) (80%). As a result, exogenous application of 10 µM Cad showed that it reduced the negative effects of drought stress on endogenous PA amounts, cell division and biochemical activities in barley.

## Introduction

Drought constitutes one of the foremost environmental variables limiting plant production on arable lands, as well as the development and productivity of plants growing under these conditions^1^. Due to the exponential expansion in worldwide population and more acute consequences of global climate change, the agricultural industry has a significant problem in delivering the food supply that people require. For these reasons, researchers are working to make plant species more resistant to stress by examining plants that show stress tolerance or sensitivity in terms of morphophysiological, biochemical, and molecular aspects^2^. Plant stress response is a complicated and highly dynamic mechanism that aims to build a new equilibrium under harsh growth circumstances. Drought, one of the most important restricting variables influencing the advancement of plants, causes changes in plant physiology by decreasing respiration, photosynthesis, and stomatal movements. Under these conditions, plants use their own specialized adaptation mechanisms to reduce the adverse consequences of drought, such as morphological and structural changes, the expression of drought-resistant genes, hormonal substance production, and osmotic regulatory substance synthesis^3^.

Polyamines (PAs) are small-molecule amines with an aliphatic structure present in eukaryotes and prokaryotes. Although the presence of putrescine (Put), spermidine (Spd) and spermine (Spm) is generally considered when PAs are mentioned in plants, thermospermine (t-spm), an isomer of Spm, and cadaverine (Cad), which has a different synthesis pathway, has also been reported in developed plants^4^. These positively charged PAs interact with proteins, nucleic acids, phospholipids that make up the membrane, and components of the cell wall to reduce damage to cells under stress^5,6^. PAs, according to earlier research, have an essential role in the regulation of defense mechanisms against environmental challenges, as well as in the physiological process and growth of plants^7,8^. PAs support normal cellular operations such as DNA replication, transcription, translation, cell formation, enzyme activity, anion-cation balance management, and membrane integrity. Furthermore, PAs play an important part in many growth and development processes, including cell division, seed germination, flower bud development, embryogenesis, fruit ripening, and response to abiotic stressors^9–11^.

Cad, which is structurally different from other PA varieties and has an independent biosynthetic pathway, is a growth regulator that acts to increase the antioxidant defense systems of plants, their tolerance to heavy metal, drought and salt stress^12,13^. Cad may enter plants through the ground in the shape of molecules generated by bacteria in the phyllosphere, rhizosphere, or endosphere, or by diffusing from decaying debris^14^, as well as being produced as a by product during the synthesis of aspartate and methionine synthesis pathway from lysine^15^. Morphological changes occur in a variety of plant species, including Arabidopsis, rice, and Scots pine, as a result of variations in plant Cad concentration caused by environmental stimuli and Cad supply from the outside^6,14,16,17^. The use of endogenous and exogenous Cad is recognized to have a vital role in plant development and stress resistance^18^. It was reported that this molecule has accumulated considerably in rape leaves^19^ under oxidative stress and pepper exposed to drought stress^20^. Furthermore, salt stress in ice plants is known to increase the amount of Cad in leaves, caused DNA damage, and increase antioxidant enzymes^21^.

Barley (*Hordeum vulgare* L.) has great economic value due to its use in both animal feed and the food industry. This crop, which is the fourth most important cereal crop in the world after wheat, maize, and rice are a cereal requered grown in drought conditions that limit plant growth due to global climate changes in our current period. Morever, it is preferred as a model plant in many experimental researches for reasons such as its easy supply, in vitro germination of seeds, small genome, and easy measurement of biochemical products such as enzymes and proteins^10,11^. As a result of our detailed literature studies, no study was found investigating the effect of exogenous Cad application on barley plants under drought stress conditions on the parameters studied here, and all the evidences obtained was presented for the first time in this study. The aims of this work are to investigated the effects of exogenous Cad application on cell cycle, endogenous polyamine amount, total protein content and biochemical parameters in barley (*H. vulgare* cv. Burakbey) exposed to drought stress. The data of this study will be a basis for accelerating the development of stress-tolerant barley varieties by providing information about the physiological, biochemical and molecular interactions in the drought stress mechanism in barley under deteriorating climates.

## Materials and methods

### Germination of seeds and plant growth conditions

Barley (*H. vulgare* cv. Burakbey) seeds used in this study were obtained from the Aegean Agricultural Research Institute. PEG 6000, which demonstrates the effect of drought stress, was obtained from Merck and Cad from Sigma. In germination experiments, exogenous Cad application was carried out by pre-soaking the seeds. Accordingly, after the seeds were kept in 50 mL of 10 µm Cad solution for 24 h, they were planted in petri dishes with 7 mL of distilled water or 22% PEG 6000. In the first stage of the study, the PEG 6000 concentration, which showed the stress effect, was determined as a result of many repeated preliminary studies. According to the results of the preliminary trials, the PEG 6000 concentration, which reduced the germination percentage below 50%, was determined as 22%. Germination experiments with sufficient amount of full-looking, robust and similarly sized barley seeds were carried out in a dark environment in the climate cabinet set at 20 °C. Before planting barley seeds in petri dishes, they were purified in distilled water for one day after soaking in a sodium hypochlorite solution for around 10 min. After these treatments were finished at room temperature, the sterilizing process was completed by drying the seeds between two filter sheets. The seeds germinated in the climate cabinet at a constant temperature of 25 °C for 7 days were planted in multipods with peat soil for seedling formation and were taken back to the climate cabinet. For 21 days, all plants in the climate cabinet were cultivated under consistent temperature and humidity settings based on the day (16 h/25 °C) and night (8 h/16 °C) cycle until seedlings were developed. Every three days, a control group of plants placed in cabinets received irrigation with 7 ml of distilled water. On the tenth day, the drought-treated plants were given 7 ml of PEG 6000. At the conclusion of the 21st day, plant population samples were extracted from the climate chamber, digested with liquid nitrogen, and employed in the experiment.

### Assay of the cell cycle

In this study, flow-cytometry was used to determine the differences between the cell cycle phases of drought stress and exogenous Cad application. The nucleic contents isolated from root tip samples collected from barley seeds germinated for 7 days were examined for cell cycle analysis. Nuclei were separated from at least three plants from each group and at least 3000 cells were counted each sample to examine changes in cell cycle stages. In sterile petri plates on ice, root tips from germinated barley were chopped into pieces using a scalpel in the presence of 5 mL of OTTO1 solution. The homogeneous mixture obtained as a result of fragmentation was filtered with 33 µm nylon mesh. After adding 10 mL of OTTO2 solution to the filtered cell mixture, analysis was performed by flow cytometry^22^. G0–G1, S, and G2 phases were similarly read in each suspension as a result of the analysis done in CyFlow Space flow cytometer (Sysmex Partec) with the aid of Flowing Software, and the findings were compared.

### HPLC analysis

HPLC analysis was performed to examine the changes caused by exogenous PAs in the amount of endogenous PAs under well watered group and drought stress. 2.5 g of fresh leaf tissue from a 21-day-old barley seedling was homogenized with a volume of 25 mL of 0.4 M HClO_4_ (perchloric acid) solution for this purpose. After centrifugation at 3 °C 10000 rpm for 10 min and filtering using a 0.45 µm filter, 400 µL of the mixture was taken, same amount (400 µL) of Na_2_CO_3_ and C_12_H_12_C_l_NO_2_S were added and incubated in a 40 ºC water bath for half an hour. Following injecting 200 µL of Sodium-l-glutamate monohydrate solution on the samples taken from the water bath, they were incubated for 1 more hour at the same temperature. Completing adding 1 mL of acetonitrile to the resultant solution and centrifuging it at 2500 rpm for 10 min, the upper stage was injected into HPLC to quantify the levels of endogenous PAs^23^.

### Total protein analysis

Ozden^24^ method was used for extraction from the leaf tissues of barley plants in order to determine the changes in the total protein amount after the application of exogenous Cad with the control group and drought stress. This procedure involved blending 300 mg of tissue from the leaves in 4 mL of potassium phosphate buffer solution containing 2 mM Na-EDTA and 1% PVP, then centrifuging it at 10,000 rpm for 10 min at 4 °C. Total protein contents were determined from the supernatants obtained as a result of centrifugation, using the Bio-Rad extraction kit, after application in accordance with the instructions, as a result of the readings made in the spectrophotometer at 595 nm^25^.

### Biochemical analysis

#### Malondialdehiyde (MDA) measurements

Li^26^ approach was utilized to quantify MDA activity with the aim to estimate the quantity of lipid peroxidation. In a mortar, 0.2 g of leaf tissue from barley seedlings was crushed with 5 mL of TCA solution and spun at 1000 rpm for 20 min. Following taking 2 mL of each concentration of the obtained supernatants, a 0.6% TBA solution in 2 mL of 10% TCA solution experienced injected. Subsequently 15 min of boiling in a steam bath, the mixture was shock-cooled on ice. Finally, the collected samples were spun at 4000 rpm for 10 min, and spectrophotometer readings at 600, 532, and 450 nm were taken. The quantity of MDA was determined using the formula C (μmol/L) = 6.45 (A_532_−A_600_)−0.56 A_450_ based on the values obtained from three distinct wavelengths.

#### Measurement of enzymes by extraction

Enzyme extraction and preparation were carried out at  4 °C temperatures to assess antioxidant enzyme levels. After rinsing the fresh leaves in 0.2 g distilled water, they were homogenized in a mill and pestle with 5 mL of cold Sodium Phosphate buffer (50 mM, pH 7.8). Homogenates were spun down for 20 min at 10,500 rpm before being stored at  4 °C for enzyme assay^27^.

#### Superoxide dismutase (SOD) measurements

Beauchamp and Fridovich^28^ approach was used to measure the amount of SOD enzyme. The mixture to be read spectrophotometrically to determine the enzyme level contains: 1.5 mL, 0.05 M Sodium Phosphate Buffer (pH 7.8), 0.3 mL, 130 mM Methionine, 0.3 mL, 750 μM NBT, 0.3 mL, 0.1 mM EDTA-Na_2_, 0.3 mL, 20 μM Riboflavin, 0.01 mL, Enzyme Extract, 0.01 mL, 4% PVPP, 0.28 mL distilled water. The resultant 3 mL mixture was placed in two 15 W fluorescent lamps for 10 min before being placed in the dark for 10 min. SOD levels were calculated by reading absorbance at 560 nm.

#### Catalase (CAT) measurements

The amount of CAT enzyme was measured using the Beers and Sizers^29^ technique. Accordingly, the mixture to be read spectrophotometrically to determine the enzyme level contains the following: 1.5 mL, 200 mM Sodium Phosphate Buffer (pH 7.8), 1 mL distilled water, 0.3 mL, 0.1 M H_2_O_2_, 0.2 mL, Enzyme extract. The CAT enzyme activity was calculated by estimating the change in the 4-min kinetic measurement of the mixture at 240 nm.

#### Ascorbate peroxidase (APX) measurements

The peroxidase enzyme activity has been assessed using Kato and Shimuzu^30^ process. The mixture to be read spectrophotometrically to determine the APX enzyme level contains: 1 mL 50 mM K-Phosphate Buffer (pH:7), 500 μL 1 mM EDTA-Na_2_, 250 μL 0.5 mM Ascorbic acid, 200 μL 0.1 mM H_2_O_2_, 50 μL Enzyme extract. The APX enzyme activity was calculated through assessing the alteration in the 4-min kinetic measurement of the mixture at 290 nm.

### Stastistical analysis

For all analyses of all parameters studied in the study, statistical findings were acquired through three replications of samples taken from three separate tissues. The SPSS program was used to identify statistical differences, and Duncan test analysis of the program was done. The results were reported as averages and standard deviations (SD), with the threshold for statistical significance set at less than 0.05. In addition Pearson Correlation Analysis and Principal Component Analysis was performed RStudio.

### Plant guideline statement

Barley (*H. vulgare* cv. Burakbey) seeds used in this study were obtained from the Aegean Agricultural Research Institute. Plant collection permits were not required because seed samples are commercial cultivar which can be purchased and no species are endangered or threatened. This study complies with relevant institutional, national, and international guidelines and legislation.

## Results and discussion

### Cell cycle densities

**Figure 1:**
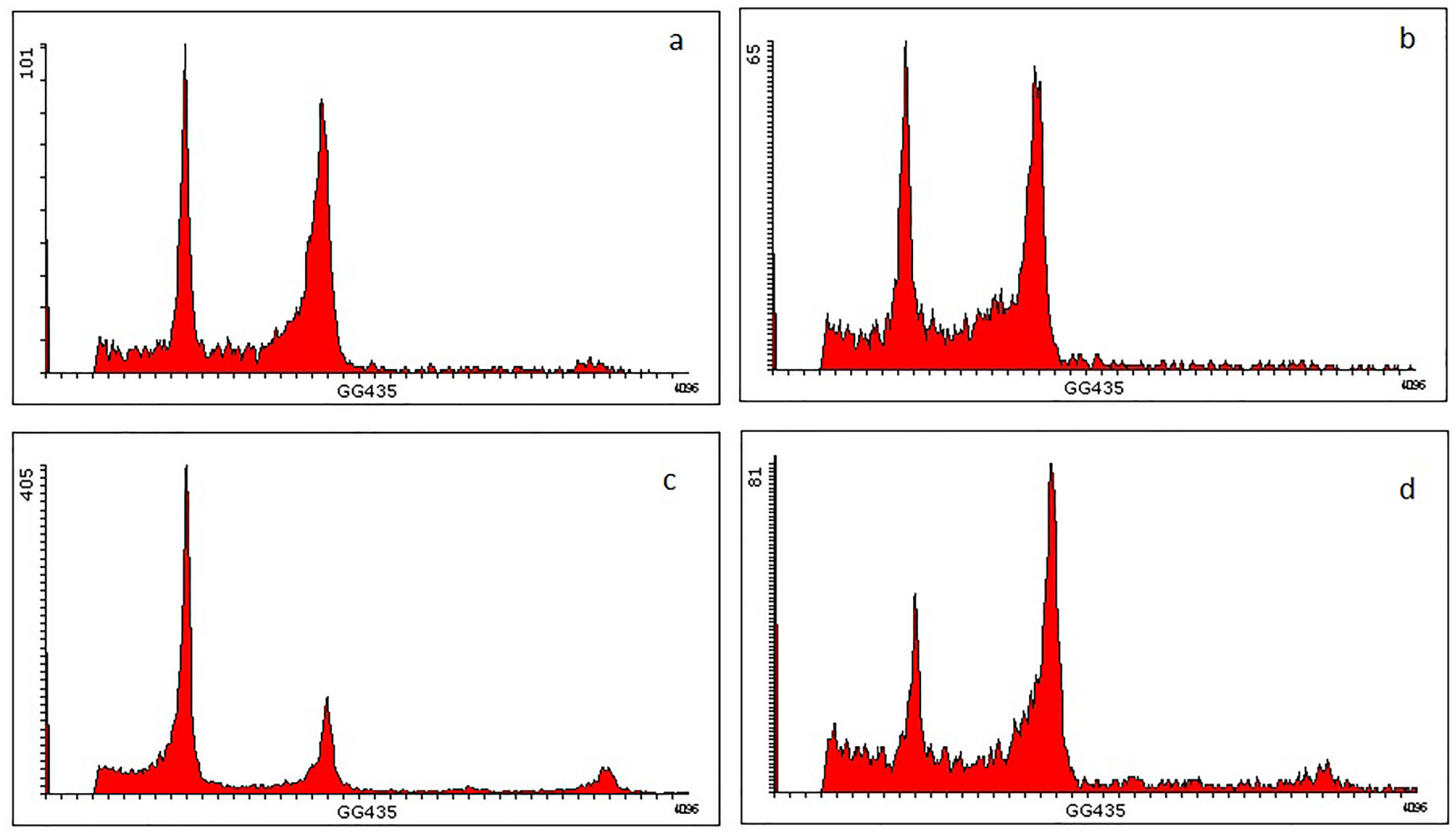
Flow cytometry graphs showing cell separation during the mitotic phase of the cell cycle in barley. (**a**) Control, (**b**) 10 µM Cad, (**c**) 22% PEG 6000, (**d**) 22% PEG 6000 + 10 µM Cad. All data were evaluated as five replicates.

**Figure 2:**
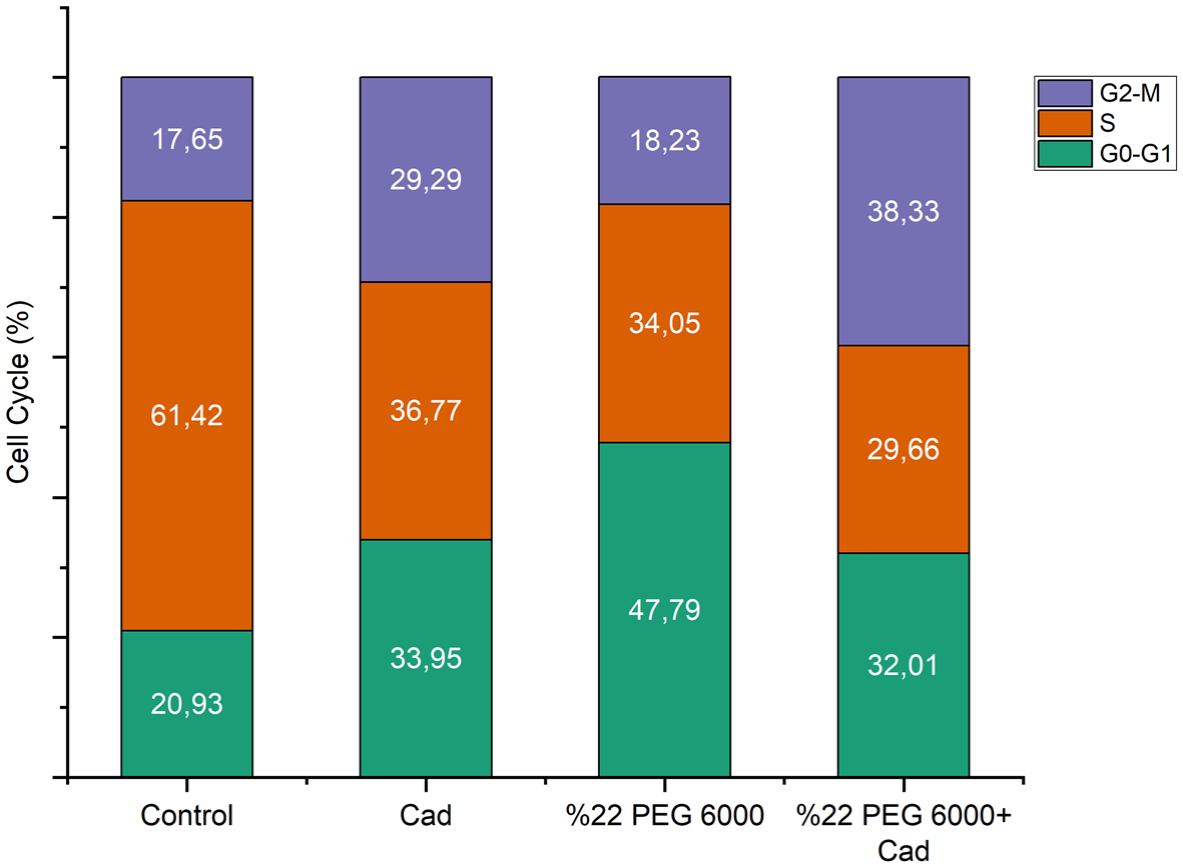
Effects of exogenous Cad (10 µM) on the cell cycle of barley seedlings cultivated under control and drought stress conditions (22% PEG 6000). The seeds were pretreated by soaking them for 24 h in consistent quantities (50 mL) of distilled water or Cad. All data were evaluated as five replicates.

Cell cycle densities of exogenous Cad application under both control group and drought stress are shown in Fig. 1. As a result of Flow-cytometry analysis in the control group, cell density was determined as 21% in the G_0_–G_1_ phase, 61% in the S phase and 18% in the G_2_–M phase of the cell cycle. Exogenous Cad application applied to the stress-free environment increased the cell densities in the G_0_–G_1_ and G_2_–M phases by more than 60%, while it decreased it by 40% in the S phase. Drought stress reduced cell density in the G0–G1 phase (130%) while increasing cell density in the S phase (45%). In the G2–M phase, cell density levels in the drought stress and control groups were quite similar. Exogenous Cad treatment produced alterations in all stages of the cell cycle during drought stress. The most noticeable of these alterations is a more than 100% rise in the G2–M phase, along with the promising impact of a drop in cell density in other stages (Fig. 2).

Plants modify intracellular molecular pathways to respond to abiotic stressors by regulating the cell cycle in real time. Cyclins, cyclin-dependent kinases, and regulatory subunits govern cell cycle phase transitions. Drought stress disrupts the cell cycle by blocking the complex structures generated by cyclin-dependent kinases and cyclins^31^. For this reason, in order for the plant to maintain its vital activities, the inhibitory effect caused by drought stress must be balanced with cell cycle control and adaptation to dynamic environmental conditions. The growth of plant tissues and organs occurs in two ways. The first is the increase in the number of cells that occurs through cell division, and the second is the expansion stage, where the cells take their final form. The cell shrinkage and reduction in cell division that occur under dry circumstances in sunflower are caused by deterioration in the cell cycle’s G_1_/S transition^32^. It has been stated in previous studies that drought stress disrupts the balance in cell cycle stages. Drought stress, according to Setter and Flannigan^33^, hampered the S stage of the cell cycle in maize, whereas the G1 and G2 stages slowed cell division due to the stress-induced increase in cell division. Furthermore, drought stress reduces CDKA activity in wheat, resulting in higher density of cells in the G1 and G2 phases of the cell cycle in reactions to detrimental effects stress^34^. This study found that drought stress increases the G_1_ and G_2_ phases of the cell cycle while decreasing the S stage in barley plants, which is consistent with other findings in the literature. There has been almost no investigation on the impact of exogenously given Cad on cell cycle during drought stress. Exogenous Cad administration in the drought-resistant barley cultivar (*H. vulgare* cv. Bülbül-89) lowered cell density in the G_1_ and G_2_ phases while increasing cell density in the S phase under drought stress, according to Özmen et al.^35^. When the impact of exogenous Cad treatment on cell densities in *H. vulgare* cv. Burakbey plant, a different barley variety, was investigated in this study, a decrease in the G_0_–G_1_ and S phases and an increase in the G_2_/M phase were discovered. The cell cycle progresses as a result of the creation of a complicated structure between cyclins, which have 7 types formed by 60 cyclin genes, and cyclin-dependent kinases (CDKs). It is thought that D-type cyclins, one of the seven types, control the transition from G1 to S phase, A-type cyclins control the transition from S to M phase, and B-type cyclins control the transition from G_2_ to M phase^32^. It is thought that exogenous Cad application under drought stress improves cell density by regulating cell cycle stages and this is due to the promotion of A, B and D type cyclins. As a consequence of this research, the influence of exogenous Cad treatment on cell cycle during drought stress were explained, and a gap in the literature in this area was filled.

### Endogenous PAs content

**Figure 3:**
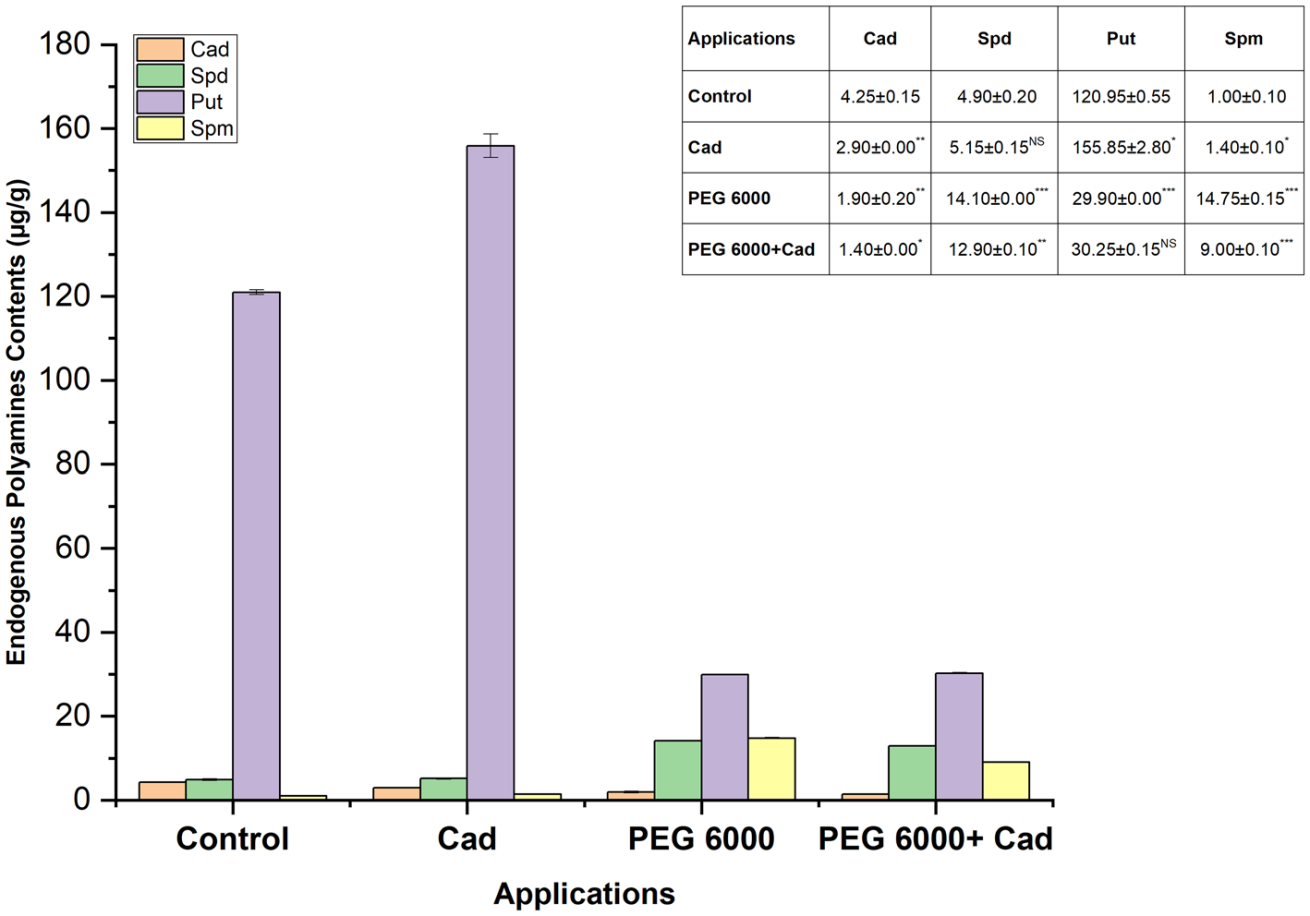
Endogenous PA content alterations in response to exogenous Cad (10 µM) on barley seedlings grown in control (distilled water) and drought stress (22% PEG 6000) conditions. The seeds were pretreated by soaking them for 24 h in consistent amounts (50 mL) of distilled water (control) or Cad. ± Standard deviation. *The difference between values; NS, no significant, *p < 0.05, **p < 0.01, ***p < 0.001. All data were evaluated as three replicates.

The changes of exogenous Cad application on the amount of endogenous PAs at all concentrations are shown in Fig. 3. When the endogenous PA amounts of the control group were examined, it was determined that the amount of Put (120.95 µg/g) was considerably higher than all other PA types. With the amount of 1 µg/g, Spm was the least abundant PA type among the endogenous PAs, while the endogenous amount of Cad and Spd, which are among the other PA types, was determined as 4.25 and 4.90 µg/g, respectively. In the absence of drought stress, it was determined that exogenous Cad application caused an increase in the endogenous amounts of Spd, Put and Spm, and a decrease in the amount of Cad. Drought stress affected the endogenous amount of all PAs differently and these changes were quite impressive compared to the control group. Drought stress caused a decrease in the amount of Cad and Put, and an increase in the amount of Spm and Spd. Among these changes, the 75% decrease in the amount of Put and an increase of approximately 13 times the amount of Spm are the highest changes in the amount of endogenous PA. The decrease in the amount of endogenous Cad, Spd and Spm and the increase in the amount of Put in response to stress after Cad applied under drought stress conditions formed a stress response mechanism. After the application of Cad in stressful environment, the endogenous PA amounts were determined as 1.40 µg/g for Cad, 12.90 µg/g for Spd, 30.25 µg/g for Put and 9 µg/g for Spm.

PAs are involved in many biological processes throughout the life cycle of plants^36^. By interacting with positively charged PAs, proteins, DNA, RNA and phospholipids, they affect basic cellular processes such as gene expression, translation, cell cycle regulation, membrane stabilization and modulation of cell death^37^. Like other types of PAs Cad, whose synthesis is different from other PA species, is involved in alleviating stress in plants under various biotic and abiotic stresses^38^. In addition, since Cad is known to have effects on the accumulation of other PAs, its contributions to the stress response cannot be evaluated alone. Instead, comparing the effects of exogenous Cad application on the endogenous amounts of other PA species under the same conditions will help to explain the effect of this PA more accurately^18^. When the studies conducted considering the specified conditions are examined, exogenous administration of Cad, which has an important role in the production of secondary metabolites^15^, caused increased Put and Spd but reduced Spm content in Arabidopsis, which does not contain Cad endogenously^16^. The results obtained from the studies show that there is a mutual interaction in the content of endogenous Cad and Put in plants during root growth^37^. As a result of this study, it was determined that exogenous Cad application to the control group in barley leaf tissues increased the amount of internal PAs in all PAs except Cad, and the relationships between the synthesis of PAs were revealed. It is thought that the increase in the amount of Put, Spm and Spd in exogenous Cad application is due to the encouragement of the enzymes and genes involved in the synthesis stages of these PAs. It has been stated by researchers that drought stress affects endogenous PA contents in different ways. Li et al.^39^ found that drought stress increased the amount of endogenous Put and Spm and decreased the amount of Spd in creeping bentgrass plants. In another study by Li et al.^40^ they revealed that drought stress in wheat clover plant, this time, decreased the amount of endogenous Put and Spm, and increased the amount of Spd. It is known that drought stress increased the amount of all PAs studied in *Zea mays*^41^, while drought stress increased the internal Spm and Spd amounts and decreased the amount of Put and Cad in the barley variety Bülbül-89^10^. The results obtained from this study were similar to the effects of drought stress on the endogenous PA contents of different barley cultivars studied previously. There is no study in the literature on the application of exogenous Cad under drought stress. The fact that the application of exogenous Cad under the stress obtained in this study causes a decrease in the amount of endogenous Cad, Spd and Spm and an increase in the amount of Put has been demonstrated for the first time. This study revealed how the endogenous PA contents were affected by the exogenous application of Cad, one of the PAs that have an important role in the response mechanism to drought stress, and extremely effective results were obtained in terms of eliminating the deficiency in the literature on this subject.

### Total protein content

**Figure 4:**
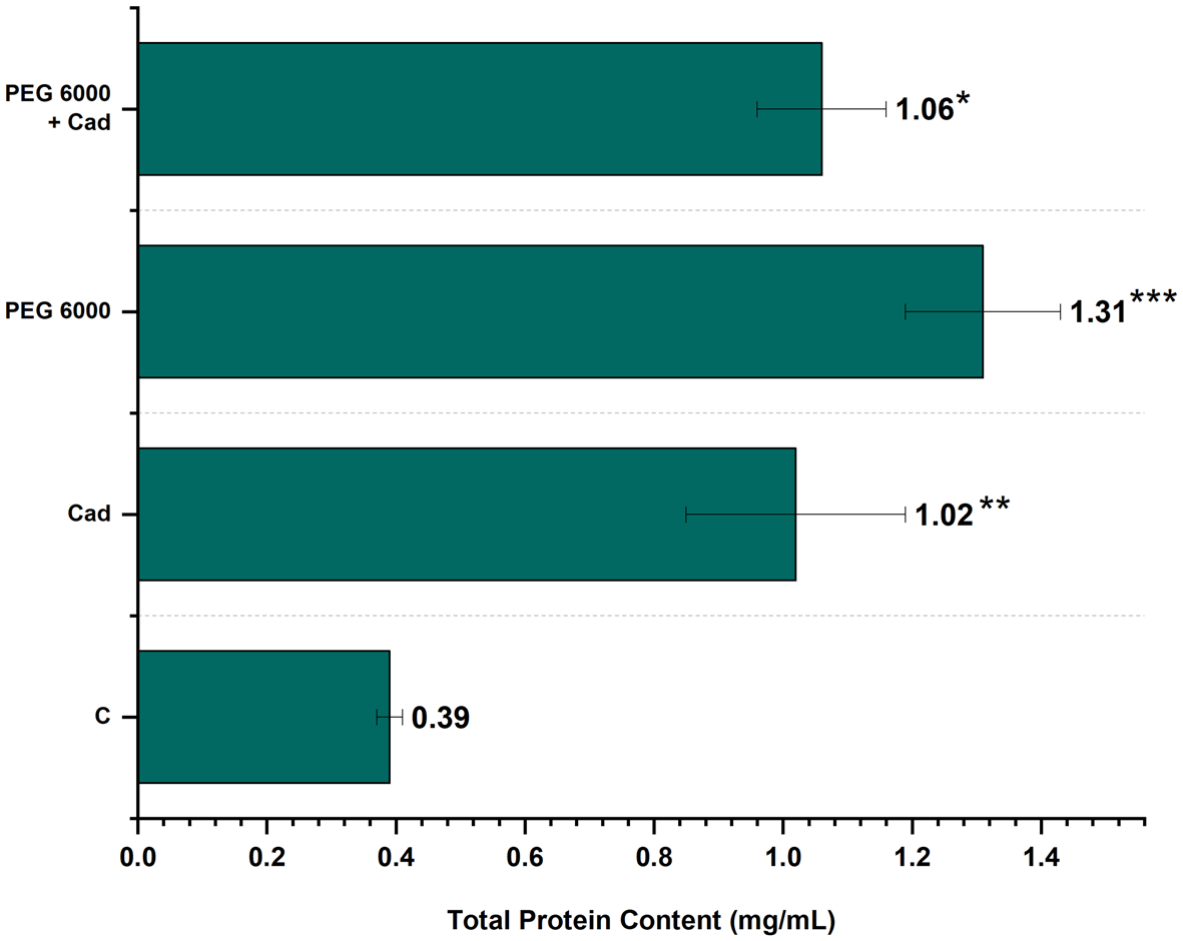
Total protein change graph of exogenously applied Cad (10  µM) in barley plant in control group and drought conditions (22% PEG 6000). The seeds were pretreated by soaking them for 24 h in consistent amounts (50 mL) of distilled water (control) or Cad. *The difference between values; *p < 0.05, **p < 0.01, ***p < 0.001. All data were evaluated as three replicates.

The changes of exogenous Cad application on the amount of total protein content at all concentrations are shown in Fig. 4. The total protein content in the control group was determined to be 0.39 mg/mL in this investigation. Application of Cad alone caused an increase of the total protein amount approximately 2 times. When the quantity of total protein following drought stress was studied, it was discovered that the amount of total protein grew three times and was at 1.31 mg/mL. Exogenous Cad administration under drought stress reduced total protein quantity by 20% as in comparison with the stress control group. The values of the total protein quantity of Cad applied in stressful and non-stressful conditions were near to each other as a result of this investigation. In other words, exogenous Cad administration mitigated the growing effect of drought stress on total protein quantity.

PA and its metabolic byproducts are known to operate as intracellular signaling molecules in plants during pathogen assaults, fruit aging prevention, and environmental stress resistance. PAs modulate enzymes and proteins required for DNA replication, transcription and translation, both under the above-mentioned and under normal conditions^42^. The rise in ROS due to drought stress leads to protein oxidation and ends with a process up to cell death. PAs give an advantage for protein homeostasis under stress situations by contributing to the preservation of cells' physiological processes against abiotic stressors due to their antioxidative characteristics and architectures that enable protein antiaggregation^43^. Previous research has shown that the total protein content of drought stress has various impacts depending on the plant species studied. Accordingly, researchers observed a reduction in total protein content in plants owing to the application of drought conditions in cotton, tomato, wheat and pistachio^44–47^, whereas other researchers reported a rise in total protein content in peanut and rice^48,49^. Although there have been several research on the total protein quantity of Spd, Spm, and Put, which are other forms of PAs under drought stress circumstances^50–52^, nearly no investigations on the application of exogenous Cad have been conducted. Özmen et al.^35^, as a result of their study on drought-resistant barley cultivar, stated that exogenous Cad application increased the total protein content even more under drought stress. Contrary to previous study, the application of exogenous Cad showed a reducing effect on the total protein content in the Burakbey variety, which can be described as drought sensitive after this study. Plant regulatory and defense systems are connected to total protein accumulations as a way to respond to environmental obstacles such as drought stress. The difference in total protein content produced by drought stress, together with Cad administered during normal and drought stress circumstances, was acknowledged in this study as an indication of the plant's cellular response to stress sensing. As can be understood from the studies carried out, drought stress differs depending on the plant species used and the overall protein content. It is obvious that PAs applied under drought stress improve the protein content of plants under stressful conditions. Although it is well established that PAs are extremely beneficial under drought stress circumstances, the dearth of studies addressing the impacts of total protein content on Cad is a significant limitation. The effects of exogenous Cad application on total protein content under normal and drought circumstances were evaluated as a consequence of this study, and this gap in the literature was filled.

### Biochemical enzyme quantities

**Figure 5:**
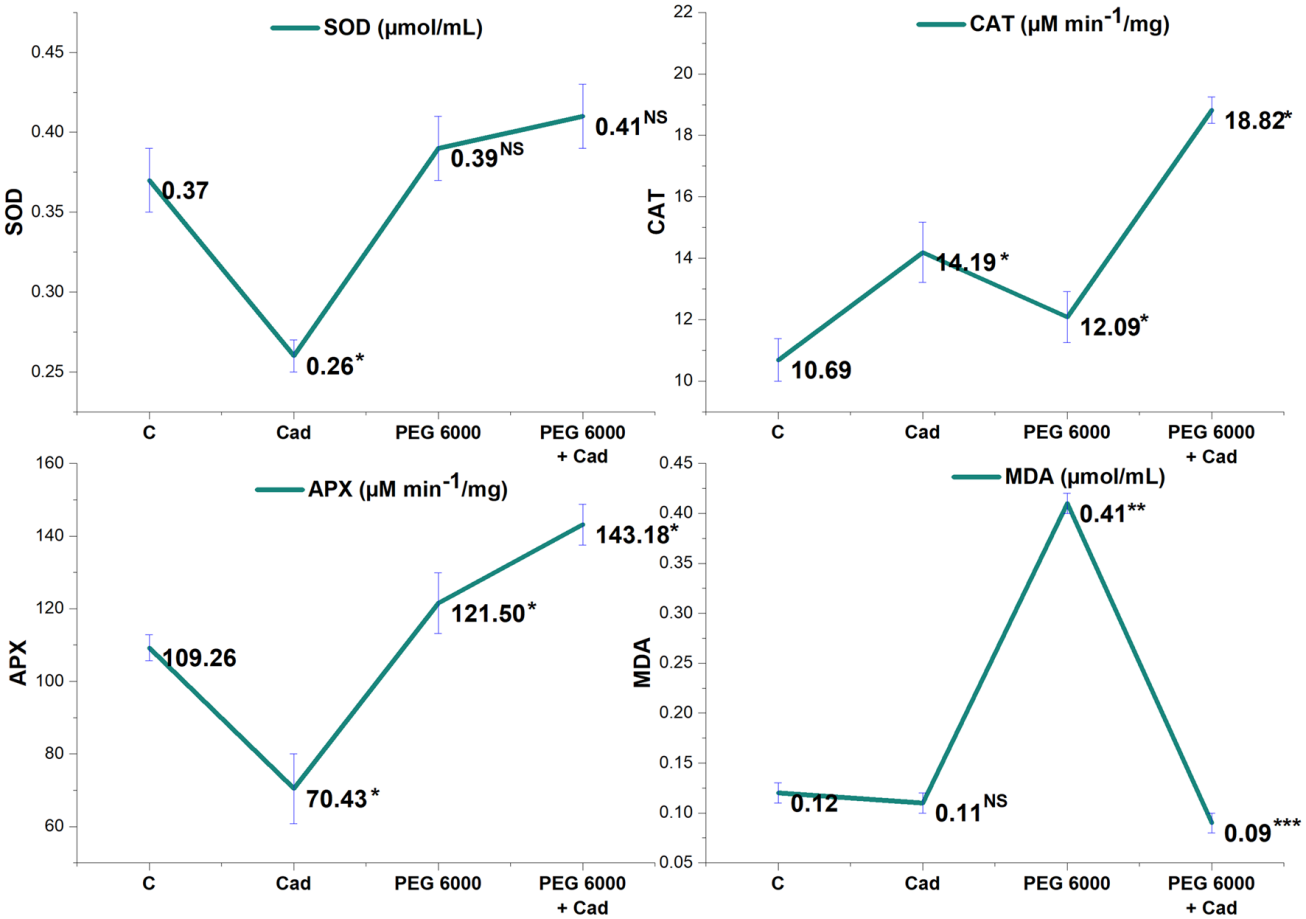
Change graph of biochemical enzymes in barley plant with exogenously applied Cad (10 µM) in control group and drought conditions (22% PEG 6000). The seeds were pretreated by soaking them for 24 h in consistent amounts (50 mL) of distilled water (control) or Cad. *The difference between values; NS, no significant, *p < 0.05, **p < 0.01, ***p < 0.001. All data were evaluated as three replicates.

Biochemical enzyme changes of Cad application under drought stress in barley genotype are given in Fig. 5. SOD, MDA, CAT and APX enzyme values of the control group were 0.37 µMol/mL, 0.12 µMol/mL, 10.69 µMol/min mg and 109.26 µMol/min mg respectively. After the application of drought stress, the amounts of SOD (5%), MDA (240%), CAT (13%) and APX (11%) increased. Cad applied to the stress-free environment increased the amount of CAT (33%), while it decreased the amount of SOD (30%), MDA (8%) and APX (35%) comparison to the group with distilled water. Except for MDA, all other biochemical enzymes increased after the administration of exogenous Cad during drought stress. While the CAT enzyme attained the greatest proportionate rise with an amount of 18.82 Mol/min mg, representing a 55% increase, the 17% change in APX enzyme level, with a value of 143.18 Mol/min mg, is noteworthy. The most striking change was the approximately 80% decrease in the amount of MDA enzyme caused by the application of Cad in drought stress.

Increases in MDA, SOD, CAT, and APX levels in barley leaf tissues show that oxidative stress is being created in the cell. MDA, a byproduct of unsaturated fatty acid peroxidation in cells, is a good marker for determining the degree of stress damage in plants^53^. Previous research has shown that the MDA concentration rises in direct proportion to the degree of plant damage in response to biotic and abiotic stimuli^54^. As a result, forecasting MDA content may be utilized to understand plant stress situations in real time and enable preventive interventions against the detrimental impacts of drought stress. According to studies carried out to date, drought stress produces a rise in MDA concentration in tomato, barley, mustard, and numerous other crops^55–57^. This research looked at the rise in MDA levels in barley leaf tissues following drought stress was similar to previous research, and this increase is believed to be caused by breakdown of the cell membrane and oxidative damage. Exogenous Cad application under drought stress improved the MDA level by 80% compared to drought stress, resulting in a great improvement in the deterioration of plant cells. SOD and CAT are enzymes which have crucial functions in the antioxidant mechanism of defense in biological systems against free radical damage. SOD converts superoxide and singlet oxygen radicals, which are produced as a result of processes in plant tissues or cells, to hydrogen peroxide and oxygen. The enzyme catalase, found common in peroxisomes, converts H_2_O_2_, which is poisonous when it accumulates in plant tissues and cells, into water and molecular oxygen, minimizing the harm that results from free radicals^58^. Researchers in diverse plants revealed enhanced SOD and CAT enzyme activity in several tests done under stress circumstances^59–62^. The rise in the number of CAT and SOD enzymes in barley leaf tissues under drought stress is connected to the fact that plant metabolism has a defensive mechanism against free radicals that increase in response to drought stress with the goal to reduce damage, according to this study. The increase in both SOD and CAT enzyme levels following the administration of external Cad pursuant to conditions of drought stress demonstrated the importance of Cad in protecting the plant under drought stress. Under stress circumstances, APX, a crucial enzyme for ROS removal in plants under biotic and abiotic challenges, is even more stimulated^63^. Melatonin-mediated modulation of the Ascorbate–Glutathione (AsA GSH) cycle is an intracellular balancing process that removes ROS generated by stress caused by drought. Because APX functions as an electron giver in the AsA cycle, it reduces the buildup of stress-induced H_2_O_2_ in the plant. Thanks to this feature, the harmful chemical accumulation is reduced with the help of APX, and the plant's stress response mechanism is formed and the plant is able to overcome this damage with the least damage^64^. Several investigations have shown that APX levels in various developed varieties of plants rise under drought stress^65–67^. Following the investigation, it was discovered that there was an increase in the level of APX in the barley leaves under drought stress circumstances, indicating that the plant had established a defensive mechanism. Cad application under stress, like other biochemical enzyme contents, increased the plant defense mechanism and increased the amount of APX even more. Although researchers use various chemicals, growth regulators and bacteria to alleviate or eliminate the negative effects of biochemical enzyme contents against stress^61,68,69^, there is no study on how exogenous Cad application affects these enzymes under drought stress. Exogenous Cad treatment was found to be effective in alleviating the effects of drought stress on all enzymes tested in this study. By revealing the stimulating properties of Cad on enzymes that are of such importance in the plant stress mechanism, the deficiency on this subject in the literature has been eliminated.

### Correlation and principal component analysis

**Figure 6:**
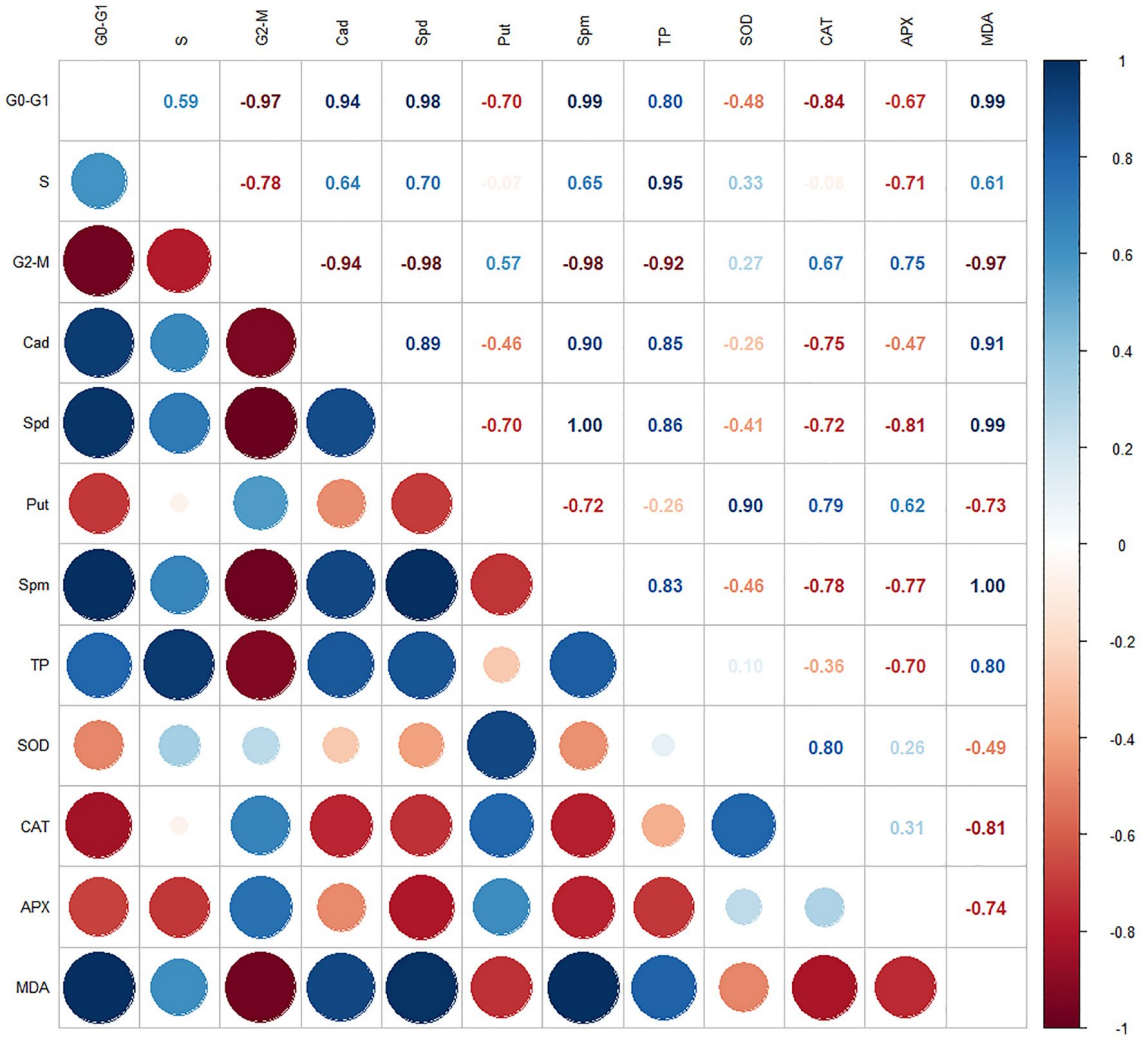
Correlation analysis values of all examined parameters formed by the effect of exogenous Cad (10 µM) applied under drought stress (22% PEG 6000) in barley plant. All data were evaluated as three replicates. According to the color bar, red color indicates negative correlation value and blue color indicates positive correlation value. G0–G1, S, G2-M: cell cycle phases; TP: total protein content; Spm: spermine; Spd: spermidine; Put: Putressine; Cad: cadaverine; SOD: superoxide dismutase; CAT: catalase; APX: ascorbate peroxidase; MDA: Malondialdehyde.

**Figure 7:**
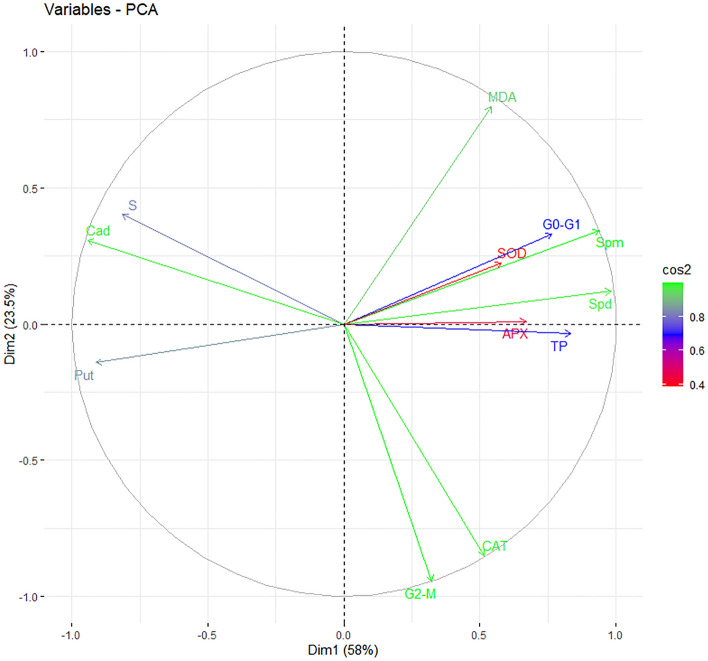
Principal Component Analysis Biplot graph between all examined parameters created by the effect of exogenous Cad (10 µM) applied in barley plant under drought stress (22% PEG 6000). All data were evaluated as three replicates. Parameters that have a high contribution to the PC1 and PC2 axes are shown in green. The first and second primary components are the horizontal and vertical axes respectively. G0–G1, S, G2-M: cell cycle phases; TP: total protein content; Spm: spermine; Spd: spermidine; Put: putressine; Cad: cadaverine; SOD: superoxide dismutase; CAT: catalase; APX: ascorbate peroxidase; MDA: malondialdehyde.

While the correlation and principal component analysis of all factors evaluated in this study are given in Figs. 6 and 7, the parameters with positive correlation are highlighted in blue, while those with negative correlation are highlighted in red. The color intensities in the circles on the left and bottom of the figure are directly proportional to the correlation coefficient, and these values are also indicated as numerical values in the right and upper parts of the figure. The first and second components formed after applying Cad under drought stress are 58% and 23.5% of the data variability, respectively (Fig. 7). According to Pearson correlation coefficients, the amount of endogenous Cad was significantly correlated with antioxidant enzymes, total protein amount, cell cycle and other endogenous PA types. It was determined that there is a very strong positive relationship between the G0–G1 phase of the cell cycle, the amount of MDA, endogenous Cad, Spd, Spm, and a very strong negative relationship CAT enzyme and G2–M phase. There was a significant positive correlation with total protein amount in S phase and negative correlation with APX enzyme and G2–M phase. The G2–M phase, the last phase of the cell cycle, has been found to have a very significant negative association with endogenous Cad, Spd, Spm, total protein content, and MDA enzyme. After the analysis, it was determined that the endogenous PA amounts were in significant relationships with all parameters in general. For example, with endogenous Cad, Spd, Spm, MDA; endogenous Spd, Spm, total protein amount showed a very strong positive correlation with MDA. However, it is clearly understood in Fig. 6 that the investigated biochemical enzymes are related to each other and to other parameters. After the analyzes applied in the study, it confirms that Cad applied under drought stress creates a resistance mechanism as a result of the interaction of the molecular and physiological parameters examined in the study. These results revealed that exogenous Cad application under drought conditions was closely associated with not only endogenous PA amounts but also biochemical enzyme activities and cell cycle changes.

## Conclusions

In this study, the effects of Cad, a type of PAs involved in the drought stress mechanism, on cell cycle, internal PA amounts, total protein and biochemical enzyme activities in well-irrigated and dry conditions were investigated. Drought stress applied to barley plants caused statistically significant changes in all parameters examined and once again revealed how stress negatively affects plant metabolism. 10 µm Cad applied under drought stress promoted cell division by increasing G1–S and S–G2 cell transitions in the cell cycle. In addition, exogenous Cad helped plant metabolism to approach its natural state by promoting the reduction of endogenous Spd, Cad and Spm amounts. Also, it enabled the formation of a stress response mechanism by reducing the amounts of Cad, TP and MDA and increasing the levels of SOD, CAT, APX enzymes. In summary, the information obtained from this study can contribute to research efforts to increase drought resistance and improve yield sustainability in barley and similar grain species exposed to drought stress.

## Data Availability

The datasets used and analysed during the current study are available from the corresponding author on reasonable request.
